# Case Report: Genetic Alterations Associated with the Progression of Carotid Paraganglioma

**DOI:** 10.3390/cimb43030159

**Published:** 2021-12-17

**Authors:** Vladislav Pavlov, Anastasiya Snezhkina, Dmitry Kalinin, Alexander Golovyuk, Anastasiya Kobelyatskaya, Ildar Bakhtogarimov, Nadezhda Volchenko, George Krasnov, Anna Kudryavtseva

**Affiliations:** 1 Engelhardt Institute of Molecular Biology, Russian Academy of Sciences, 119991 Moscow, Russia; vladislav1pavlov@gmail.com (V.P.); kaa.chel@mail.ru (A.K.); bakhtogarimov@gmail.com (I.B.); gskrasnov@mail.ru (G.K.); 2 Vishnevsky Institute of Surgery, Ministry of Health of the Russian Federation, 117997 Moscow, Russia; dmitry.v.kalinin@gmail.com (D.K.); algolovyuk@inbox.ru (A.G.); 3 National Medical Research Radiological Center, Ministry of Health of the Russian Federation, 125284 Moscow, Russia; mnioict@mail.ru

**Keywords:** carotid paraganglioma, recurrent tumor, metastasis, *SDHB*, *TERT*, whole-exome sequencing, case report

## Abstract

Paragangliomas (PGLs) are rare neuroendocrine tumors that can develop from any paraganglion across the body. The carotid body is the most often location of PGLs in the head and neck region. Carotid PGLs (CPGLs) are characterized by predominantly non-aggressive behavior; however, all tumors have the potential to metastasize. To date, molecular mechanisms of paraganglioma progression remain elusive. We report a case of a 38-year-old woman with metastatic CPGL manifesting as a recurrent tumor with lymph node metastasis. The tumor was fast-growing and had a high Ki-67 proliferation index. Immunohistochemical (IHC) examination and whole-exome sequencing were performed for both recurrent tumor and metastasis. A germline pathogenic splice acceptor variant in the *SDHB* gene was found in the patient. Immunoreactivity of the SDHB subunit was weak diffuse in both samples, indicating deficiency of the succinate dehydrogenase. Moreover, the recurrent tumor exhibited loss of heterozygosity (LOH) at the SDHB locus, that is according to Knudson’s "two-hit" hypothesis of cancer causation. We also identified a rare somatic promotor mutation in the *TERT* gene associated with the tumor progression. Obtained results confirmed the indicative role of the germline *SDHB* mutation for metastatic CPGLs, as well as the potential prognostic value of the *TERT* promoter mutation.

## 1. Introduction

Head and neck (HN) paragangliomas (PGLs) are rare neuroendocrine tumors that form from the paraganglia of the parasympathetic nervous system. HNPGLs are classified as carotid, vagal, middle ear (jugulotympanic), and laryngeal depending on their localization [1], but can also occur at other rare sites of the head and neck [2]. Carotid paragangliomas (CPGLs) are the most common type of HNPGLs that account for about 60%. They manifest as a predominantly painless slow-growing neck mass located at the bifurcation of the carotid artery [3]. CPGLs can occur as bilateral (10–25%) and multiple (10%) tumors [1]. Metastatic cases are officially diagnosed in 4–6%; however, all CPGLs have the potential to metastasize [1]. 

HNPGLs can develop both as hereditary or sporadic tumors. Hereditary HNPGLs may be a part of the paraganglioma syndromes (PGLs) 1-5 caused by germline mutations in the following genes: *SDHD* (PGL1), *SDHAF2* (PGL2), *SDHC* (PGL3), *SDHB* (PGL4), and *SDHA* (PGL5) [4]. There are other susceptibility genes, such as *VHL, RET, NF1, TMEM127, MAX, FH,* and *SLC25A11,* that may also contribute to hereditary HNPGLs [5]. Mutations in these genes predispose phenotypic differences at the molecular level (molecular clusters) and clinical manifestations [6]. Thus, a mutation in the *SDHB* gene is associated with a high risk of malignancy when *SDHD* mutation often results in multiple tumors [7]. The evidence for other malignancy biomarkers such as variants in the *TERT* promotor is also arising [8,9]. However, despite advances in the genetics of PGLs, one of the main challenges remains the identification of markers for malignancy risk. Nowadays, the determination of metastatic PGLs is based on the presence of metastasis. Data on potential markers for diagnosis of metastatic PGLs and prediction of aggressive tumor behavior are limited and controversial, meanwhile it plays an important role in the management of the disease. 

We present a case of a 38-year-old woman harboring metastatic CPGL with metastasis in a regional lymph node that was surgically removed and studied using whole-exome sequencing. Analysis of genetic changes occurring in tumors and metastasis within a patient can improve understanding of potential mechanisms underlying the tumor progression.

## 2. Case Presentation

A 38-year-old woman was referred to the Vishnevsky Institute of Surgery, Ministry of Health of the Russian Federation with a recurrent tumor on the left side of the neck. At medical history, she reported neck swelling and left side neck mass diagnosed at 18 years old by herself. She described the fast growth of the neck mass during a year, which was then removed in a local hospital. Seventeen years after the surgery, a fast-growing neck mass developed on the same site. The tumor irregular in shape was surgically resected with the adjacent lymph nodes. The larynx and digastric muscle were adjacent to the tumor medially, dystrophic sternocleidomastoid muscle—laterally, spinous processes of 2–3 cervical vertebrae—from behind. The tumor went beyond the corner of the lower jaw, adjoining the lower pole of the parotid gland, from which it was separated by an acute way.

**Figure 1:**
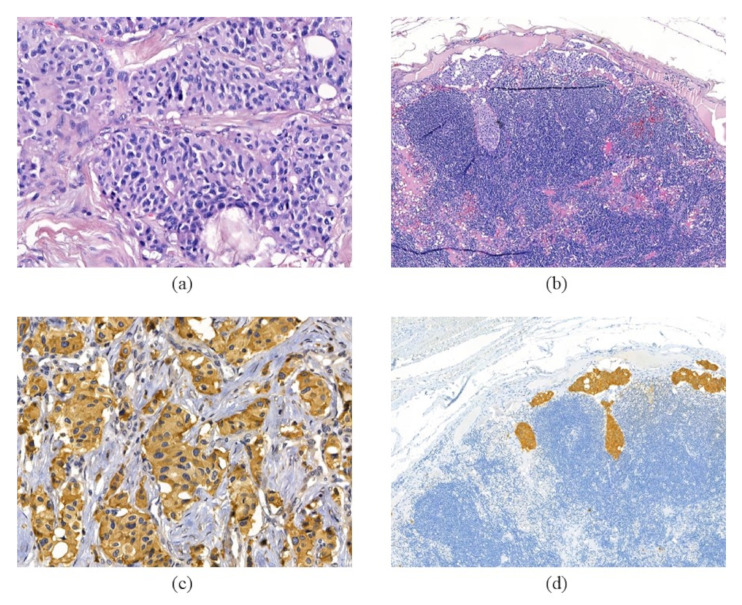
Histologic and immunohistochemical sections of recurrent tumor and metastasis. Hematoxylin–eosin staining of the tumor (**a**) and lymph node (**b**) tissues displays a specific “Zellballen” growth pattern. Chromogranin A antibodies stain chief cells in the tumor (**c**) and metastasis (**d**).400× magnification for (**a**) and (**c**) and 100× magnification for (**b**) and (**d**).

Histological and morphological examination of the resected recurrent tumor confirmed carotid paraganglioma (CPGL) (Figure 1, Supplementary Materials Figure S1). The tumor was sized 37 × 30 ×45 mm, had a predominantly solid structure, and was characterized by vascular invasion and invasion of surrounding tissue. Hematoxylin–eosin (H & E) staining showed a Zellballen structure that is typical for paragangliomas. Chief tumor cells exhibited positive staining for chromogranin A and synaptophysin indicating a neuroendocrine tumor. S100 protein was expressed in sustentacular cells. The result of immunostaining for cytokeratin AE1/AE3 was negative. Histological examination also revealed metastasis in an adjacent lymph node with the same structure and expression of neuroendocrine markers (Figure 1, Supplementary Materials File S1—Figure S1). The Ki-67 proliferative activity in the recurrent tumor was 20%.

**Figure 2:**
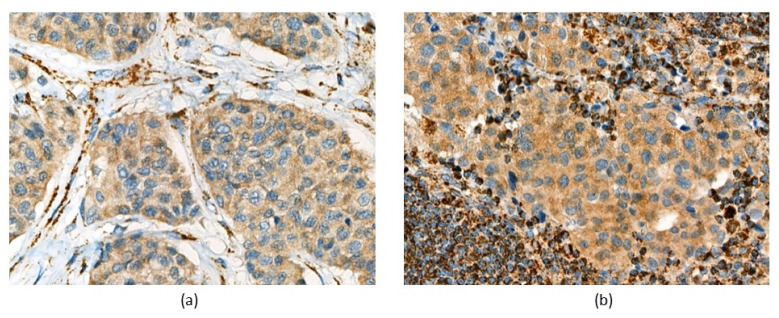
Immunohistochemical examination showed weak diffuse SDHB staining in (**a**) recurrent tumor and (**b**) metastasis of the patient. Magnification 400×.

Immunohistochemistry (IHC) analysis of four succinate dehydrogenase subunits (SDHA, SDHB, SDHC, and SDHD) was performed for both tumor and metastasis. IHC staining was carried out and interpreted as described in [10]. Both samples showed weak-diffuse SDHB staining (Figure 2). All reactions for SDHA, SDHC, and SDHD were immunopositive (Supplementary Materials File S1—Figure S2).

Genetic testing was performed for the patient after obtaining informed consent using whole-exome sequencing. The DNA from tumor, metastasis, and normal lymph node tissues were extracted with a High Pure FFPET DNA Isolation Kit (Roche, Switzerland). Exome libraries from all three tissue samples were prepared using a KAPA HyperExome (Roche, Switzerland) and sequenced on an Illumina NextSeq 500 System (USA) with paired-end mode (76 × 2). Analysis of sequencing reads was performed as previously described [5] with several improvements. Bowtie 2 was used for alignment and the latest version of GATK4 (v. 4.2) was applied for base quality score recalibration. Variant calling was performed with the GATK pipelines, HaplotypeCaller and Mutect2, for germline and somatic variant detection, respectively. The pathogenicity of variants was predicted by calculation of “pathogenicity score plus” based on the population frequency, site conservation, summary weighted score across several prediction algorithms, and clinical significance.

**Figure 3:**
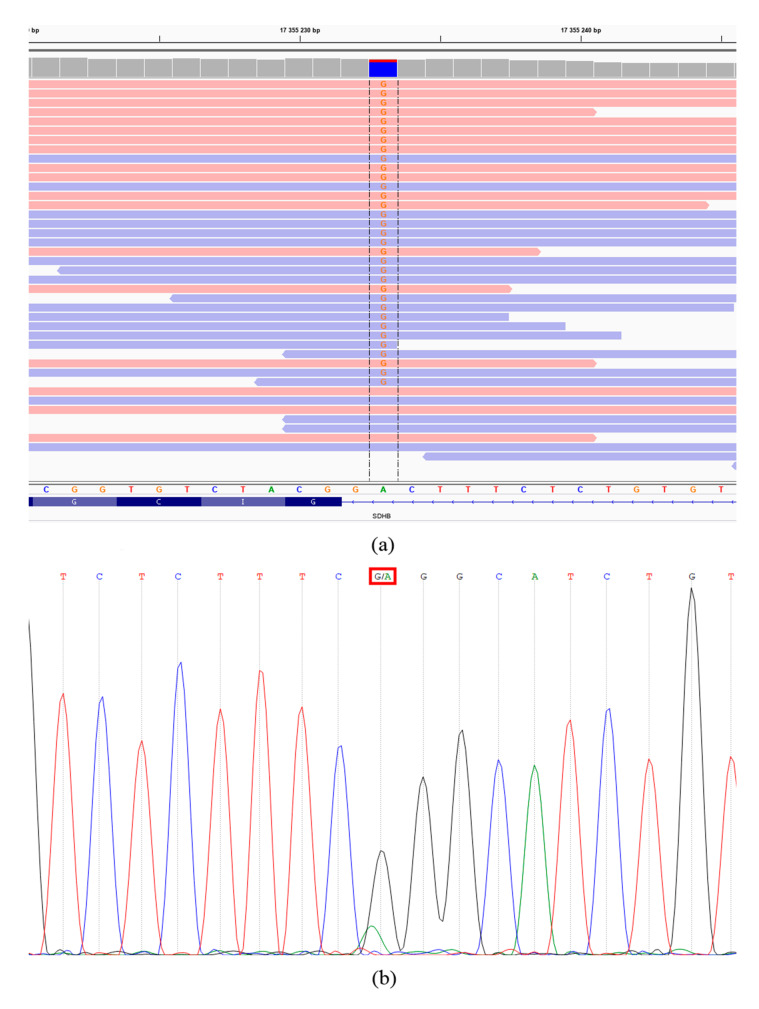
Validation of the SDHB c.287-2A>G mutation with Sanger sequencing in the recurrent tumor. (**a**) Exome sequencing data (variant nucleotides are marked by red color, forward and reverse reads present as pink and blue horizontal lines, respectively). (**b**) Sanger sequencing chromatogram.

Exome analysis revealed a germline pathogenic variant NM_003000: c.287-2A>G (chr1: 17355233, rs1064794270) in the *SDHB* gene affecting an acceptor splice site at the 3′ end of an intron 3. The result was verified in the tumor using Sanger sequencing (Figure 3). According to the ClinVar database and based on the criteria of the American College of Medical Genetics and Genomics and the Association for Molecular Pathology (ACMG-AMP) [11], the variant was classified as pathogenic. This germline mutation has been previously found in one familial paraganglioma [12] and two HNPGLs (described as 421-2A>G) [7]. Buffet et al. also identified this germline variant among a large cohort of patients with PPGLs subjected to genetic testing [13]. We did not find any germline variants in other HNPGL susceptibility genes.

**Figure 4:**
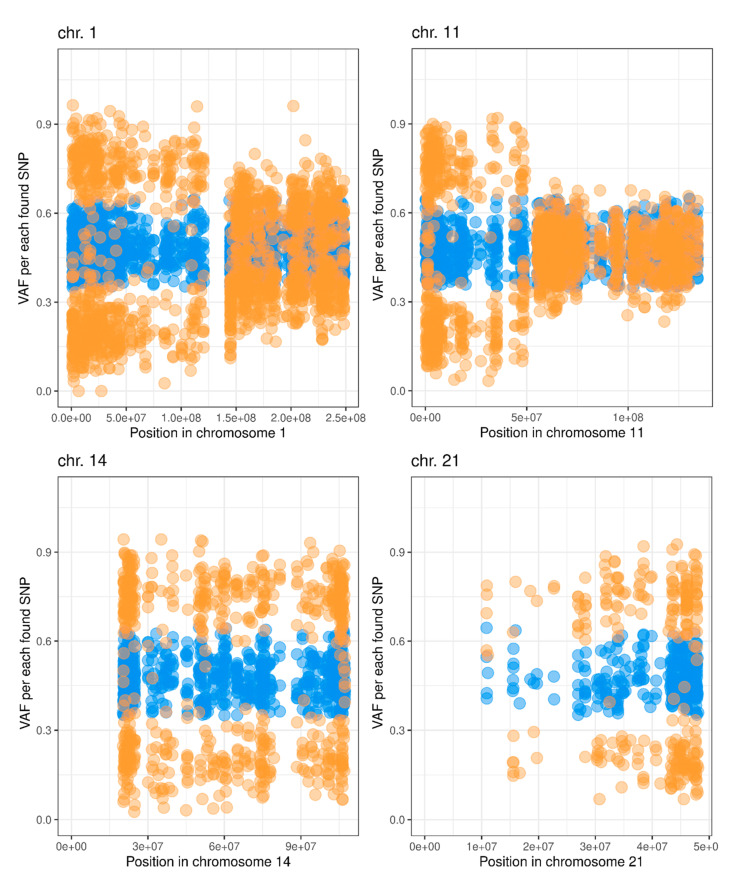
Variant allele frequency (VAF) across chromosome 1, 11, 14, and 21 of recurrent tumor (orange dots) and lymph node (blue dots) in a patient with CPGL.

To identify copy number variations (CNVs) in tumor and metastasis, we performed a simple comparative (tumor vs. lymph node) beta allele frequency (BAF) analysis in the following way. First, using variant calling data for the matched normal tissue, we choose heterozygous single nucleotide polymorphisms (SNPs) that: (a) have total read depth > 25 for both normal and tumor samples, (b) have variant allele frequency (VAF) from 0.35 to 0.65, and (c) are annotated in dbSNP (v. 150). Next, we compared VAF values between the tumor and matched normal samples using the exact Fisher’s test separately for each SNP. The tumor-normal difference between VAF values (delta-VAF) greater than 0.2 or less than –0.2, which passed Fisher’s test *p* < 0.05 threshold, were considered as significant. Finally, per-chromosome VAF and delta-VAF plots were generated (Supplementary Materials File S1—Figures S3 and S4). The same analysis was applied for the comparison of metastasis vs. the lymph node (Supplementary Materials File S1—Figures S5 and S6). As a result, deletion of the p-arm of chromosomes 1 and 11, as well as loss of chromosomes 14 and 21 were revealed in the recurrent tumor when no alterations were found in the metastasis sample (Figure 4).

We also analyzed somatic mutations and mutational load in the recurrent tumor and metastasis. Somatic missense mutations in the *TOR1AIP1*, *KRT33B*, *COMMD2*, *ZNF367*, and *TERT* were found in both tumor and metastasis samples (Supplementary Materials File S2). Missense variant in the *HAT1* gene was observed only in metastasis. All variants were characterized by low population frequency (≤0.01), high conservation score (PhastCons and PhyloP), and were predicted as deleterious by many prediction tools. Variants in most genes were previously detected in other cancers (Cosmic v. 70, v. 90, and ICGC). Additionally, we calculated weighted mutational load (wML) depending on VAF according to the previously reported algorithm [14]. We analyzed wML by three modes: “metastasis/tumor vs. lymph node”, “metastasis/lymph node vs. tumor”, and “tumor/lymph node vs. metastasis” (Supplementary Materials File S2). All comparisons demonstrated high wML at low VAF that was progressively decreased when it reached 0.2 VAF. Thus, most somatic variants with VAF < 0.2 are possibly sequencing errors or FFPE artifacts. Under a setting threshold of VAF = 0.2, wML per megabase (Mb) in recurrent tumor and metastasis was 0.260 and 0.037, respectively.

## 3. Discussion

In the literature, data on the incidence of recurrent HNPGLs are limited, and factors associated with the tumor progression are unknown. Kevin et al. reported a 10.5% rate of recurrence incidence for CPGLs highly associated with metastasis development (~70%) at 10 years of follow-up [15]. In the present case, relapse occurred 17 years after the initial surgery that is in concordance with the reported median time to recurrence for HNPGLs, 18.4 years [15]. Unusually for PGLs, both primary and recurrent tumors of the patient have been characterized by fast growth. The Ki-67 proliferation index of the recurrent tumor was 20%. Several studies showed an association between a high Ki-67 index (>2%) and risk of malignancy in PGLs [16,17,18].

Metastatic CPGLs develop in approximately 4–6% of cases [1] and are associated with decreased survival of patients [19]. The risk of a metastatic tumor is greater for sympathetic PGLs of extra-adrenal localization and is significantly lower for parasympathetic HNPGLs [20]. Young age and familial disease have also been suggested to correlate with a higher risk of malignancy [21]. In the present case, the primary tumor was diagnosed in an 18-year-old woman who harbors germline pathogenic mutation in the *SDHB* gene but without any history of familial disease. The presence of the germline mutation in the *SDHB* gene was also confirmed by the IHC analysis that showed weak diffuse SDHB staining in both recurrent tumor and metastasis indicating deficiency of mitochondrial complex II [10,22,23].

Germline mutation in the *SDHB* gene is considered a key genetic marker indicating an increased risk of aggressive behavior of PGLs. The incidence of metastatic tumors in *SDHB*-mutated HNPGLs varies from 5.6% to 83% [24,25,26]. According to Knudson’s “two-hit” hypothesis, inactivation of tumor suppressor gene in hereditary tumors is caused by two hits that are germline mutation as the first one and somatic alteration leading to complete loss of wild-type allele as the second dramatic event [27]. This concept can be also applied for the understanding of PGL tumorigenesis. Burnichon et al. revealed losses of heterozygosity (LOH) of driver tumor suppressor genes, including *SDHB*, in most hereditary PGLs [28]. In our case, we found large deletion of the p-arm of chromosome 1 where the *SDHB* gene is located (Ensemble, 1p36.13). However, BAF analysis revealed LOH at the *SDHB* locus only in recurrent tumor but not in metastasis. Moreover, no somatic point mutations in the *SDHB* gene were found in metastasis. Thus, metastasis and recurrent tumor could develop from different tumor clones that have different pathways of the second *SDHB* allele inactivation. For example, promoter methylation could cause wild-type allele inactivation in metastasis cells. Metastasis was also characterized by the lower mutational load (0.037 in metastatic cells vs. 0.260 in the recurrent tumor, VAF = 0.2).

Somatic mutation profiling revealed several common variants in recurrent tumor and metastasis. Identified promoter mutation in the *TERT* gene (NG_055467.1: g.586G>A [C228T]) was previously detected in many tumors according to the ICGC database (ICGC ID MU832963). Somatic promotor mutation is a common cause of *TERT* transcription activation in cancer [29]. Overexpression of *TERT* was also shown in PGLs and was frequently correlated with malignancy [30,31]. However, *TERT* activation in PGLs was found to be rarely associated with promoter mutation (<1%) [30,32]. Despite this rarity, we found hotspot somatic promotor mutation C228T in both recurrent tumor and metastasis of the patient with CPGL indicating its importance in the disease progression. Moreover, in the present case, the C228T mutation co-occurred with the *SDHB* germline variant as in several previously reported studies on metastatic PGLs, further supporting their association in *SDHx*-related tumors [8,9,30].

The somatic missense variant in the *ZNF367* gene was earlier found in endometrioid carcinoma and melanoma (COSMIC v. 90 ID COSV64546908). Knockdown of *ZNF367* has been shown to increase invasion and migration of adrenocortical, thyroid, and lung cancer cells [33,34]. Moreover, elevated *ZNF367* protein expression was observed in malignant pheochromocytoma compared to the benign tumor and normal adrenal medulla [33]. Literature data and our findings suggest that *ZNF367* may be involved in the malignization of PGLs. Among other somatically mutated genes found in both tumor and metastasis samples, only *KRT33B* was previously observed in potential association with tumors of nervous tissue, such as glioma (COSMIC v. 90 ID COSV52441330, ICGC ID MU4718621) and ependymoma [35]. The spectrum of predicted somatic deleterious mutations in the recurrent tumor was very similar to those in metastasis; a missense variant in one gene, *HAT1*, was found in metastasis but not in the recurrent tumor. The *HAT1* gene, encoding for type B histone acetyltransferase, was shown to be widely involved in the promotion of tumorigenesis and anti-cancer drug resistance [36]. The identified variant has been previously observed in pancreatic and gastric cancer (ICGC ID MU1690271) but the effect of this variant on protein structure and function has not been assessed.

## 4. Conclusions

The rarity of metastatic PGLs makes it difficult to optimize diagnostic and treatment strategies for the disease. Metastatic PGLs are diagnosed only by the presence of paraganglionic cells in non-chromaffin organs. Despite several studies supposing a list of potential markers for the risk of malignancy, reliable predictors for aggressive tumor behavior have not been found. In the presented case of metastatic CPGL, we identified some of these molecular characteristics associated with metastasis and recurrence, such as germline mutation in the *SDHB* gene and LOH, high Ki-67 proliferation index, as well as somatic promotor mutation in the *TERT* gene. This case confirmed that these alterations may serve as potential indicators for the CPGL progression.

## Data Availability

All data generated or analyzed in this study are included in the published article. The exome sequencing data are available in the NCBI SRA under the accession numbers PRJNA749948.

## Supplementary Materials

The following are available online at https://www.mdpi.com/article/10.3390/cimb43030159/s1, Supplementary File S1: [Figure S1: Immunohistochemistry images for Hematoxylin/Eosin, AE1/AE3, Synaptophysin, Chromogranin A, and S100 protein in recurrent tumor and metastasis; Figure S2: Immunohistochemistry images for subunits of the SDH complex in recurrent tumor and metastasis; Figure S3: Variant allele frequency (VAF) across all chromosomes of recurrent tumor (orange dots) and lymph node (blue dots); Figure S4. Delta-Variant allele frequency (VAF) across all chromosomes; Figure S5. Variant allele frequency (VAF) across all chromosomes of metastasis (orange dots) and lymph node (blue dots); Figure S6. Delta-Variant allele frequency (VAF) across all chromosomes]; Supplementary file 2: Somatic variants, Mutational load.
