# Supplementary material for: Case Report: Genetic Alterations Associated with the Progression of Carotid Paraganglioma
Source: Curr Issues Mol Biol. 2021 Dec 17;43(3):2266–75. doi: 10.3390/cimb43030159 (PMC8928979; doi:10.3390/cimb43030159)
Supplement: Supplementary file 1 [file cimb-43-00159-s001.zip › cimb-1451701-supplementary File S1.pdf]

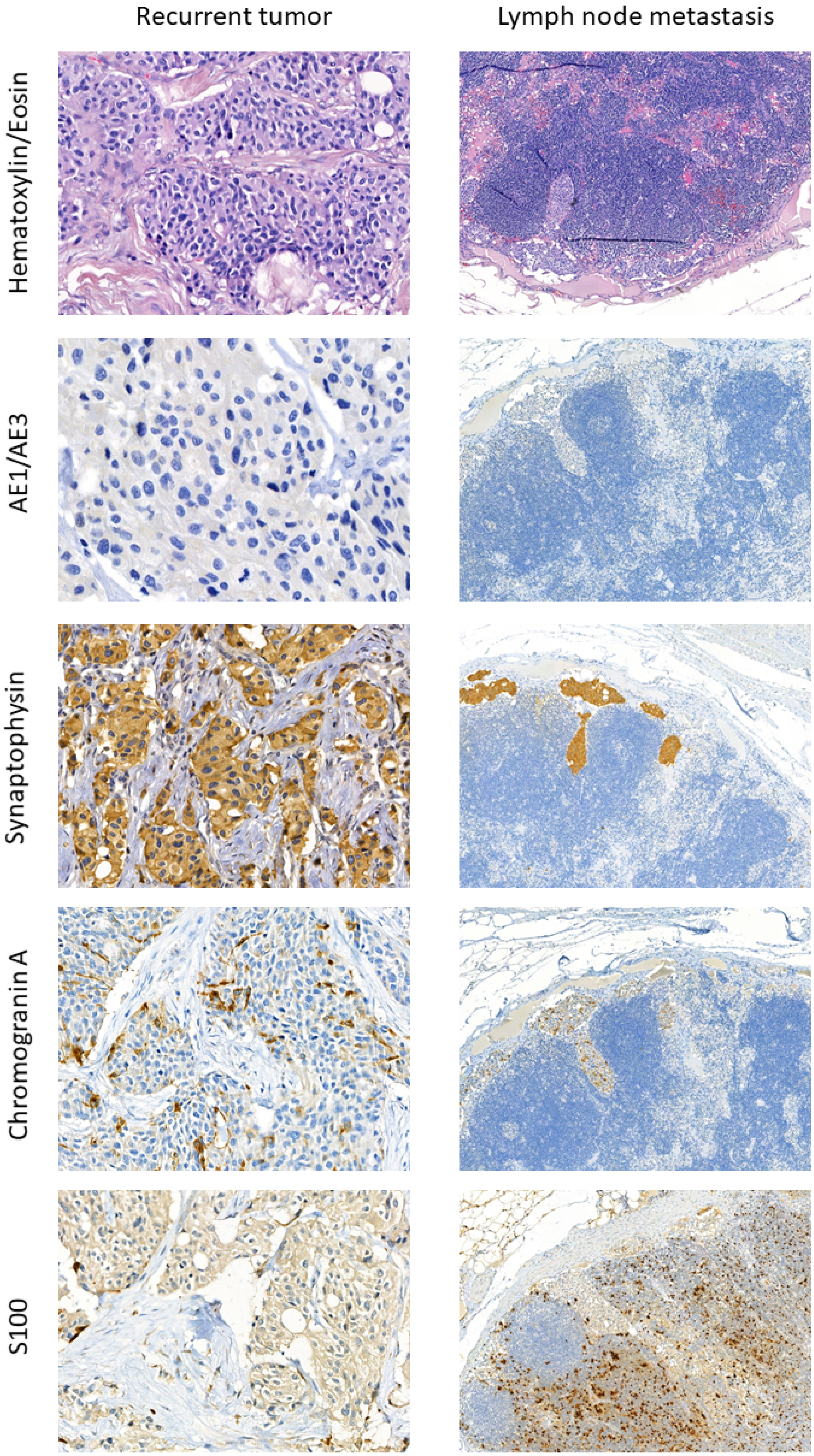

Figure S1. Immunohistochemistry images for Hematoxylin/Eosin, AE1/AE3, Synaptophysin, Chromogranin A, and S100 protein in recurrent tumor and metastasis.

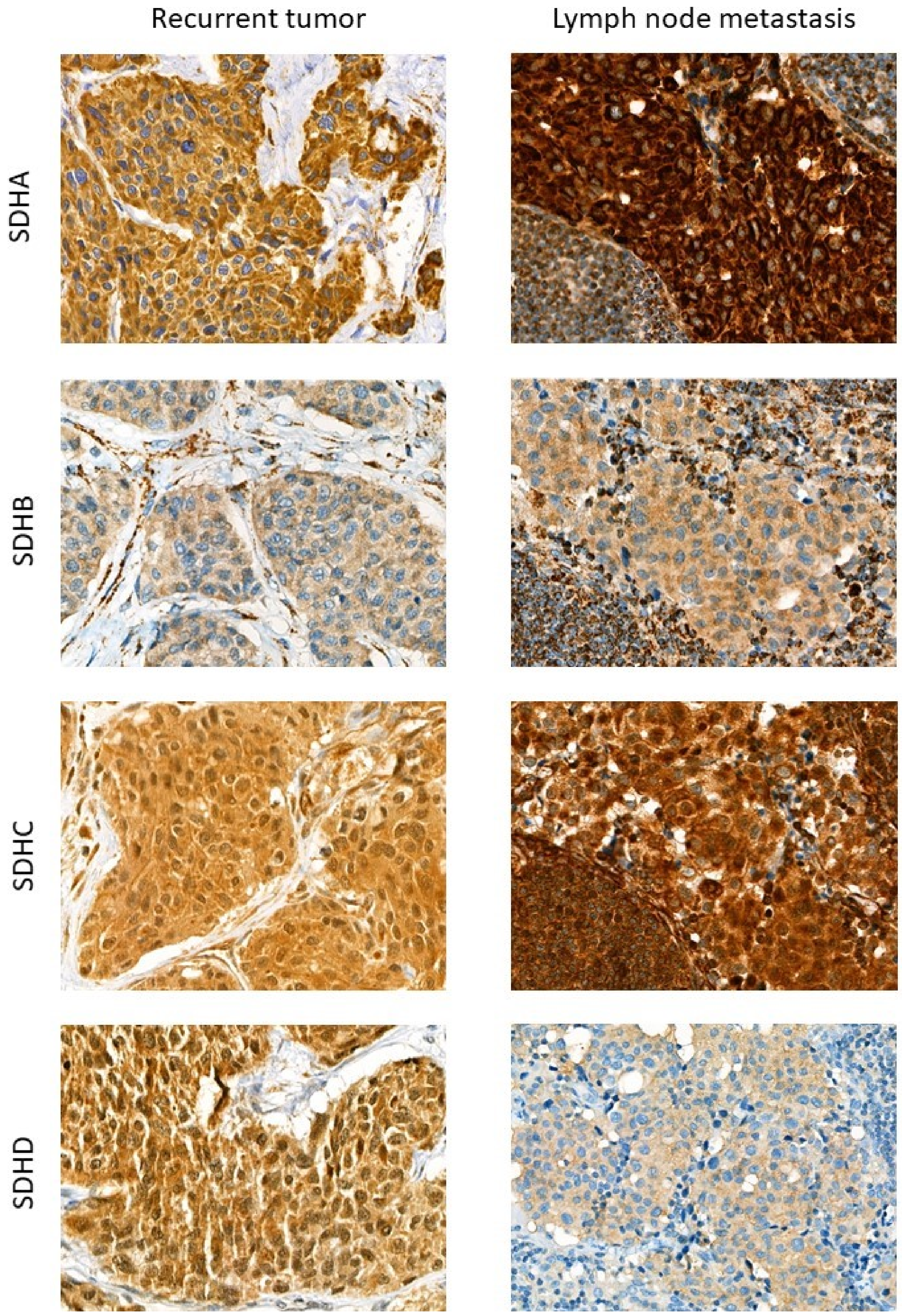

Figure S2. Immunohistochemistry images for subunits of the SDH complex in recurrent tumor and metastasis.

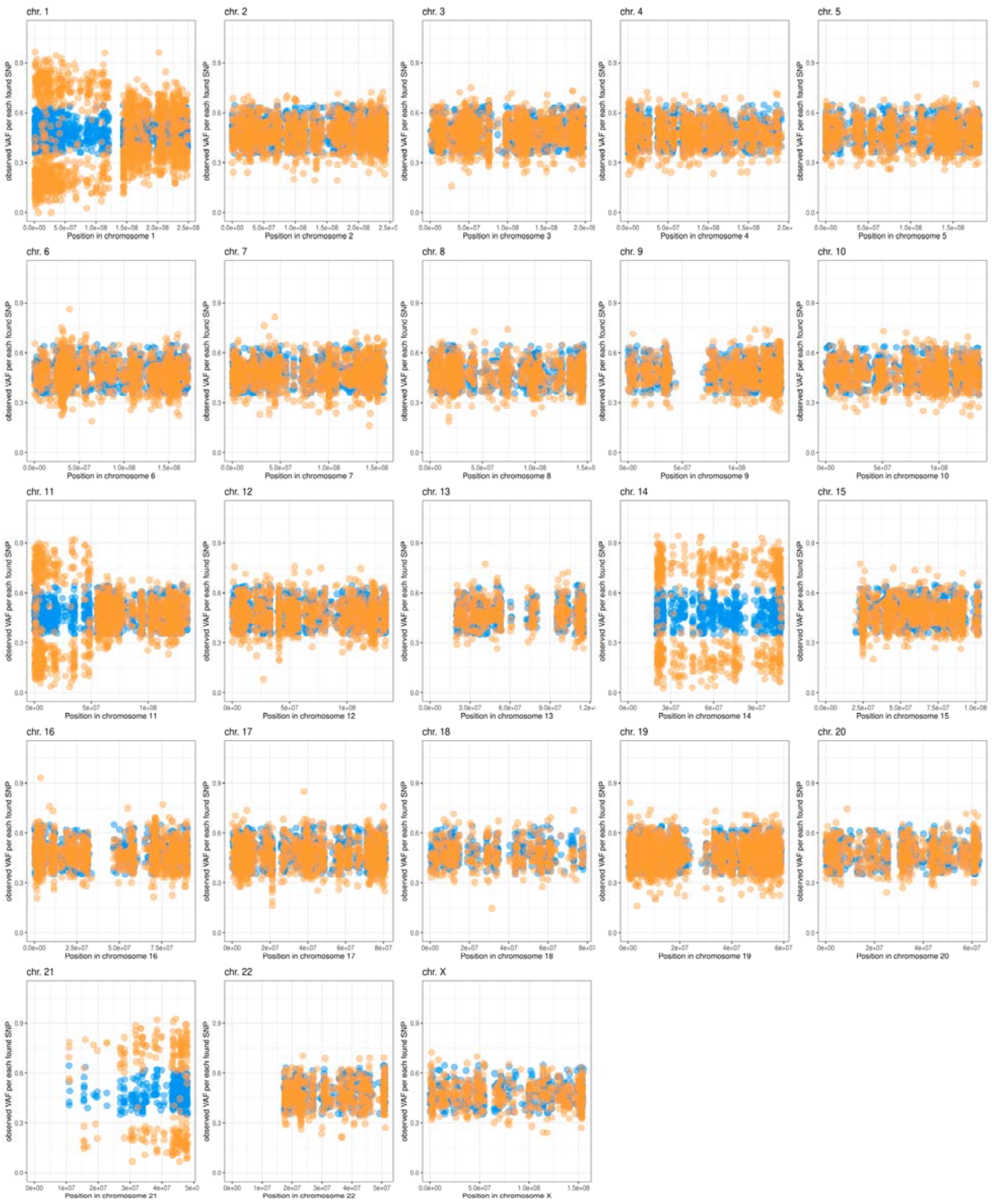

Figure S3. Variant allele frequency (VAF) across all chromosomes of recurrent tumor (orange dots) and lymph node (blue dots).

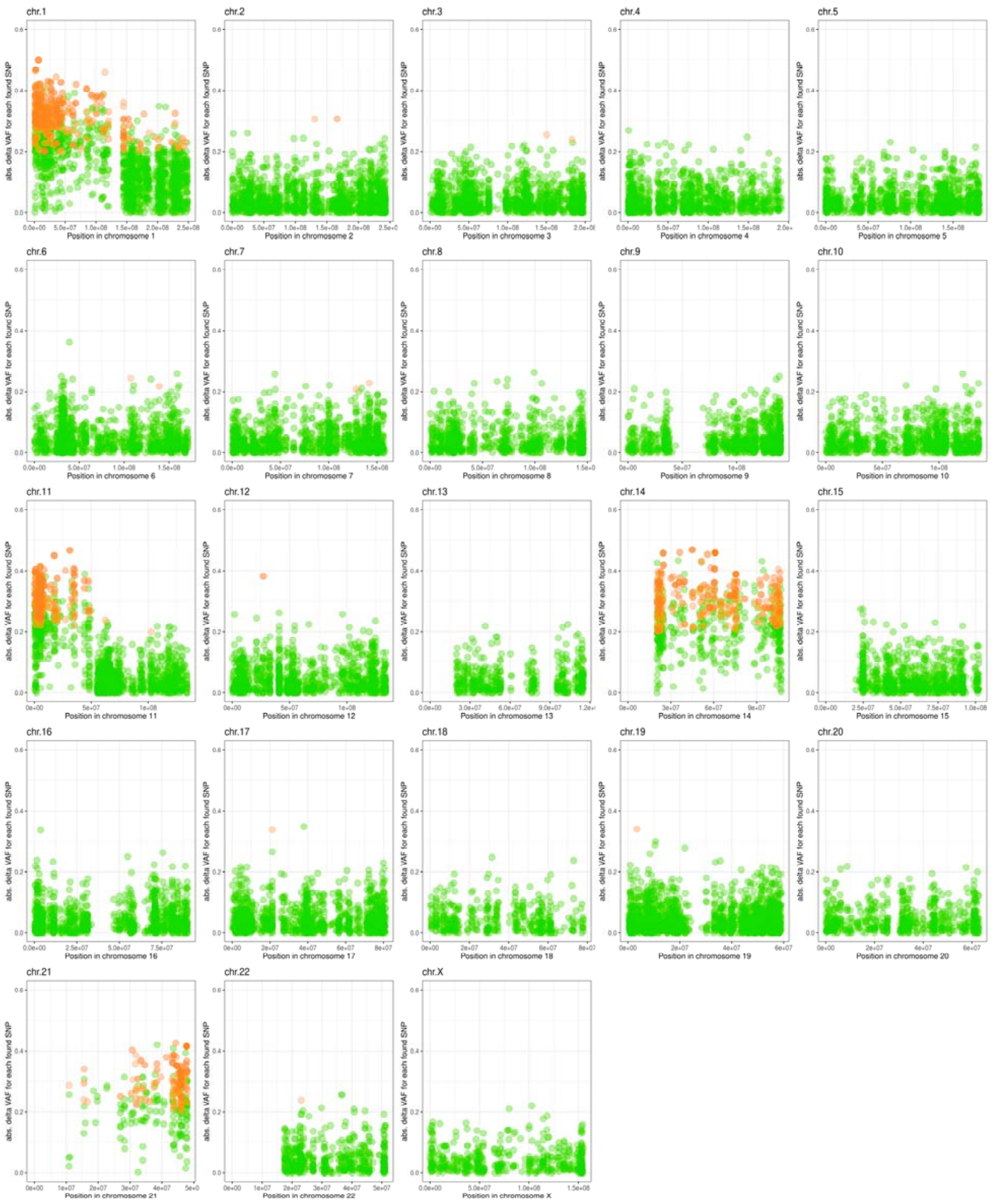

Figure S4. Delta-Variant allele frequency (VAF) across all chromosomes. Green dots indicate VAF with a difference between recurrent tumor and lymph node less than 0.2, orange dots marked those with more than 0.2.

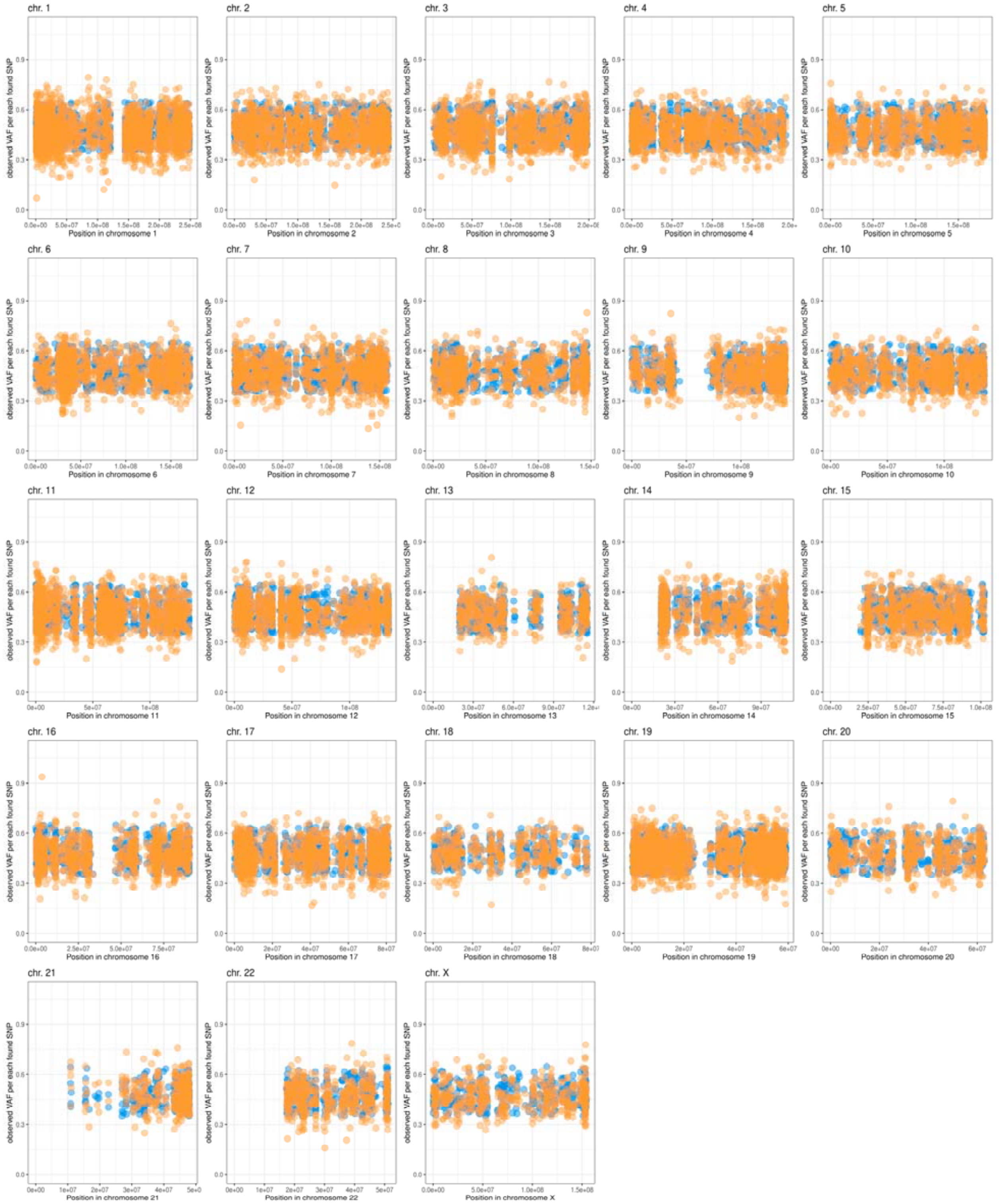

Figure S5. Variant allele frequency (VAF) across all chromosomes of metastasis (orange dots) and lymph node (blue dots).

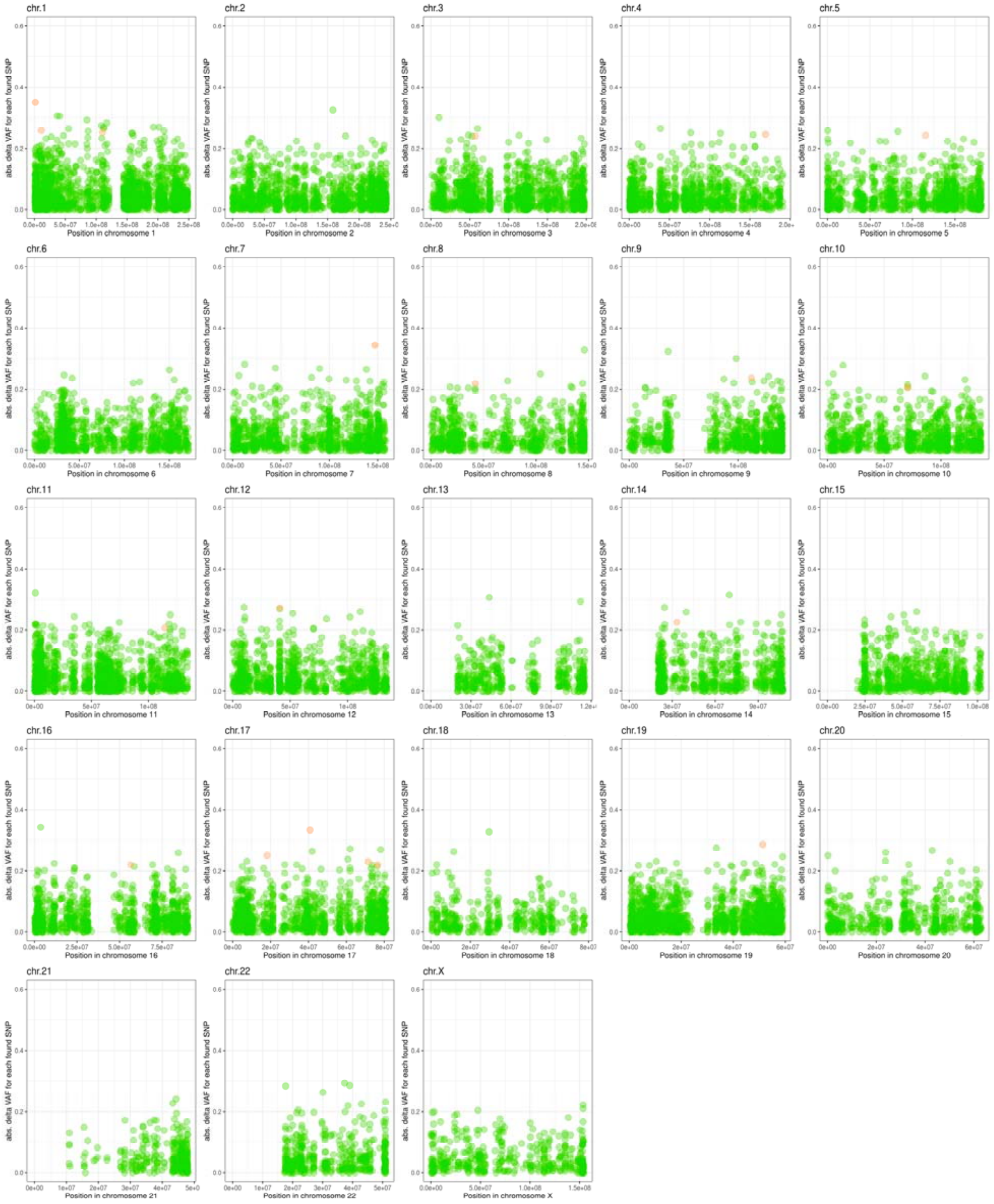

Figure S6. Delta-ariant allele frequency (VAF) across all chromosomes. Green dots indicate VAF with a difference between metastasis and lymph node less than 0.2, orange dots marked those with more than 0.2.
